# Deep Feature Extraction for Resting-State Functional MRI by Self-Supervised Learning and Application to Schizophrenia Diagnosis

**DOI:** 10.3389/fnins.2021.696853

**Published:** 2021-08-27

**Authors:** Yuki Hashimoto, Yousuke Ogata, Manabu Honda, Yuichi Yamashita

**Affiliations:** ^1^Department of Information Medicine, National Center of Neurology and Psychiatry, National Institute of Neuroscience, Kodaira, Japan; ^2^Institute of Innovative Research, Tokyo Institute of Technology, Yokohama, Japan

**Keywords:** deep-learning, functional MRI, neural network, feature extraction, psychiatric diagnosis, self-supervised learning

## Abstract

In this study, we propose a deep-learning technique for functional MRI analysis. We introduced a novel self-supervised learning scheme that is particularly useful for functional MRI wherein the subject identity is used as the teacher signal of a neural network. The neural network is trained solely based on functional MRI-scans, and the training does not require any explicit labels. The proposed method demonstrated that each temporal volume of resting state functional MRI contains enough information to identify the subject. The network learned a feature space in which the features were clustered per subject for the test data as well as for the training data; this is unlike the features extracted by conventional methods including region of interests (ROIs) pooling signals and principal component analysis. In addition, applying a simple linear classifier to the per-subject mean of the features (namely “identity feature”), we demonstrated that the extracted features could contribute to schizophrenia diagnosis. The classification accuracy of our identity features was comparable to that of the conventional functional connectivity. Our results suggested that our proposed training scheme of the neural network captured brain functioning related to the diagnosis of psychiatric disorders as well as the identity of the subject. Our results together highlight the validity of our proposed technique as a design for self-supervised learning.

## Introduction

In this study, we propose a novel deep-learning technique which extracts a feature from brain functional magnetic resonance images (fMRIs). Our proposed method solely depends on MRI-scans and does not require any additional data regarding the subjects (e.g., diseases or cognitive impairments), whereas the extracted features effectively capture the psychopathological characteristics of the subjects. Recent advances in machine learning have demonstrated its capability for medical sciences. Skin cancers have been successfully diagnosed from skin images (Esteva et al., 2017) and retinal diseases from three-dimensional optical coherence tomography (OCT) images (De Fauw et al., 2018). In addition, Titano et al. (2018) reported that machine learning with three-dimensional brain computed tomography (CT) images performed well in terms of detection of acute neurologic events including stroke, hemorrhage, and hydrocephalus. These studies suggested the further potential of the deep neural networks, especially for the analysis of spatially structured data, including MRIs and functional MRIs. These studies trained a neural network to directly infer diseases from the input. This framework is called fully supervised learning and is known to be effective when a large training dataset with accurate labels is available. Titano et al. (2018), who aimed to classify acute neurological events, collected 37,236 brain images with clinical annotations for training and used 96,303 extra clinical reports to make the clinical annotations more suitable for training. In the supervised learning framework, the network is specialized for the target diseases, which further enhances the performance. However, the requirement of a vast amount of training data is not always practical; the number of patients is sometimes too small to train a neural network (Durstewitz et al., 2019; Khosla et al., 2019), and the accurate diagnoses require expert skills (Durstewitz et al., 2019). These drawbacks are remarkable especially for psychiatric disorders because the sample size tends to be small, accurate diagnoses are especially difficult, and the underlying mechanisms are still under discussion. In contrast, self-supervised learning does not require any explicit labels for training. Instead, the teacher signals (i.e., labels) are generated from the original input data in self-supervised learning. For example, Noroozi and Favaro (2016) proposed a self-supervised learning scheme for natural image processing, in which the input image was divided into nine pieces, and the network was trained to infer the original position of each piece. The intermediate outputs of the network were subsequently fed into another linear classifier, which resulted in comparable performance to fully supervised deep neural networks. The advantages of self-supervised training potentially overcome the shortages of clean labels for psychiatric disorders, although the teacher signal must be carefully designed. In many previous deep-learning studies for MRIs without additional labels (Suk et al., 2016; Aghdam et al., 2017; Heinsfeld et al., 2018; Oh et al., 2019; Yamaguchi et al., 2021), the teacher signal was the same as the input, namely auto-encoder. Such an auto-encoder tends to suffer from the bias-variance trade-off, wherein the network either underfits or overfits the teacher signals due to a lack of constraints to the feature manifold. In contrast, this study proposes a novel self-generated teacher signal for resting-state functional MRI; we used the temporal volumes as input, and the subject ID as the teacher signal. The explicit labels enable the network to generate a compact feature that represents a conceptual distance from the owner of the input to the subjects used in the training. In this study, we experimentally showed that: (i) each temporal volume of functional MRI contains enough information to identify the subject, (ii) the network learned a feature space in which the features cluster subject-by-subject for test data as well as for training data, and (iii) the extracted feature contributes to a schizophrenia diagnosis. These experiments together exhibit the validity of our proposed method as a design for self-supervised learning.

## Materials and Methods

### Dataset

We used a dataset from the Center for Biomedical Research Excellence (COBRE) (Aine et al., 2012). The dataset is composed of anatomical and resting-state functional MRI scans; 72 scans were from schizophrenia patients and 75 from healthy controls. The anatomical and functional scans were acquired by MPRAGE and EPI by 3.0-Tesla Siemens Trio scanner (Siemens Healthineers, Erlangen, Germany). Each functional scan was composed of 150 timepoints, and the repetition time was 2 s. Each timepoint was originally composed of 64 × 64 × 32 voxels (3 × 3 × 4 mm^3^), which was transformed to 91 × 109 × 91 voxels in MNI coordinates by the preprocessing (Supplementary Section 1). We excluded subjects without meta-data and controls with other psychiatric diseases, resulting in 69 patients (56 males and 13 females, 37.8 ± 14.0 years old) and 72 controls (51 males and 21 females, 35.9 ± 11.7 years old). We divided the patients and controls into training 1, training 2, and test dataset with random sampling stratified over present illness, age, and gender. The training 1 dataset was used for training the neural network, and training 2 was used for training the linear regressor for inferring the subject attributes. The number of patients *p* and controls *c* was (*p*,*c*) = (51, 54) in training 1, (9, 9) in training 2, and (9, 9) in test datasets. The mean and standard deviation of the ages were 37.0 ± 13.4 in training 1, 36.4 ± 11.6 in training 2, and 36.3 ± 11.9 in test datasets. The number of males *m* and females *f* was (*m*,*f*) = (78, 28) in training 1, (15, 3) in training 2, (14, 4) in test datasets. We unequally allocated samples to the three datasets because the neural network in training 1 possessed a huge number of optimization parameters (about 2 million), while the linear regressor/classifier used in training 2 has a relatively small number of optimization parameters (3–10,000).

### Training 1

**FIGURE 1:**
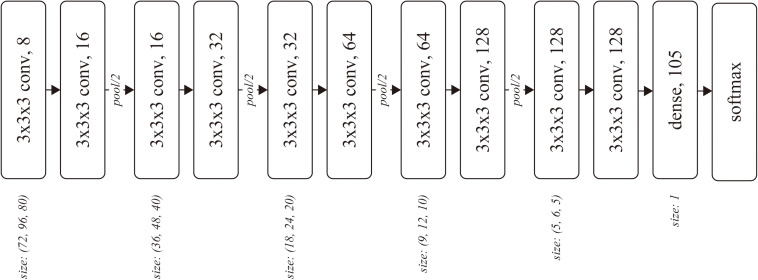
Network architecture. Each rounded square represents a layer with the weight parameters. The number after the comma denotes the number of channels for the layer.

The input of the network was a batch of temporal MRI volumes, whose size was set to (80, 96, 80) by trimming outside of the brain. The network included four convolutional blocks, followed by two convolutional layers and one dense layer. Each block consisted of two three-dimensional convolutions and one average pooling layer (Figure 1). The number of convolutional blocks was preliminarily explored. The less number of convolution blocks resulted in underfitting, wherein the training accuracy was almost the same as the chance, while the training did not converge for the network with more convolution blocks.

The kernel size *k* and stride *s* were (*k, s*) = (3, 1) for each convolutional layer, and (2, 2) for each pooling layer. The number of output channels was set as 8 at the first convolutional layer and doubled before the pooling layers, resulting in 128 before the dense layer. We used softmax cross-entropy as the loss function, which was computed against a 105-dimensional one-hot vector of subject ID. The network was optimized using Adam (Kingma and Ba, 2015) with α = 0.0001 for the first 17,000 iterations and α = 0.00001 for the following 110,000 iterations, with a batch size of 32.

### Training 2

The output of the dense layer was extracted for each timepoint as a feature vector. Subsequently, the feature vectors were averaged for each subject, yielding an identity feature for each subject. The identity features for the training 2 dataset were then fed into a linear classifier (regressor) to learn schizophrenia diagnosis and age regression.

We also trained a linear classifier with a slightly modified version of the feature vector, in which the average of all elements in the feature vector was subtracted from each element. This operation was naturally introduced by the formulation of the softmax function, in which the subtraction of the average does not affect the output of the function or the training process. In the following sections, we call the original feature vector (the output of the dense layer) as *classification*, and the modified one as *classification+*.

### Experiment 1: Training Convergence

The training accuracy of the subject classification was computed to evaluate training. Reporting training accuracy is slightly unconventional in studies on neural networks because the convergence of training is now trivial in conventional two-dimensional natural scene image processing. However, to the best of our knowledge, this is the first report which trained networks to classify the subject from a single timepoint of functional MRI by stacked three-dimensional convolutions, and we concluded that the training convergence is worth reporting.

### Experiment 2: Qualitative Analysis of Extracted Features

The characteristics of the acquired feature space were first qualitatively analyzed. We plotted the feature vectors in the training 2 and test datasets by t-distributed stochastic neighbor embedding (t-SNE) (Maaten and Hinton, 2008). The clusters were then quantitatively evaluated by precision@150 for each identity feature. Because the number of timepoints was 150 for each subject, precision@150 would be 1 if all the feature vectors for a subject clustered around his identity feature. The formula of precision@150 is given in Supplementary Section 2, wherein the feature vectors are ranked by Euclid distance to each identity feature in the original feature space. We applied these qualitative and quantitative analyses to the features of the *classification* and *classification+* feature vectors as well as the signals averaged over the ROI defined by the automated anatomical labeling (AAL) atlas (Suk et al., 2016; see Tzourio-Mazoyer et al., 2002 as the reference to AAL), namely ROI-pooled signals, and the top three and 10,000 principal components (Damaraju et al., 2014). For these features, the “identity” feature was also defined as the centroid for each subject.

We explored all the principal components up to 10,000 where the rational upper limit of computational resources for experiments 2 and 3. The results demonstrated that the number of principal components did not affect precision@150 in experiment 2 and the statistical significance of schizophrenia diagnosis in experiment 3, while the number affected the age regression performance in experiment 3 wherein the top three principal components performed the best and showed statistical significance in correlation between predicted and actual age. Therefore, we reported the results of the best (top three) and the maximum (10,000) principal components for experiment 2 and 3.

### Experiment 3: Relation to Subject’s Attributes

The schizophrenia classifier and age regressor developed in section “Training 2” were applied to the test dataset. The classification accuracy was computed and tested using a sign test, which evaluates a probability parameter of the binomial distribution underlying that the classification is significantly larger than chance (50%). This procedure was also applied to the identity features of the ROI-pooled signals, the top three and 10,000 principal components, similar to that in Experiment 2. In addition, the procedure was applied to the functional connectivity matrix (Liang et al., 2006; Kim et al., 2016), defined as the correlation coefficients among the time series of the ROI-pooled signals.

### Ethics Statement

All experiments in this study were performed in accordance with the Ethical Guidelines for Medical and Health Research Involving Human Subjects in Japan.

## Results

### Experiment 1: Training Convergence

The network was trained to classify the subject ID from each time point of fMRI. The training accuracy at the 127,000 iteration was 97.85%, which was considerably improved over the chance rate, suggesting that the training successfully converged.

### Experiment 2: Qualitative Analysis of Extracted Features

**FIGURE 2:**
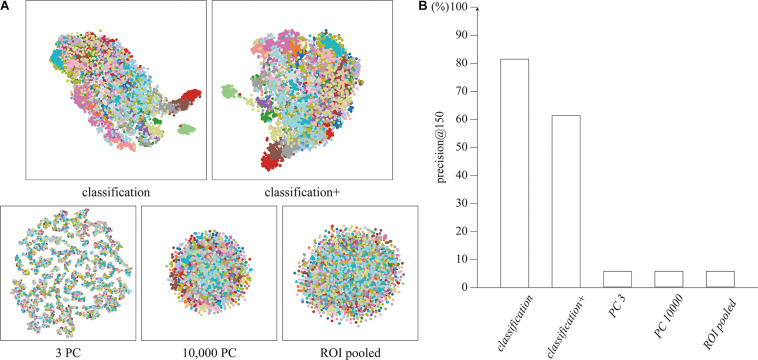
**(A)** Distribution of feature vectors, visualized by t-SNE. Each dot represents a feature vector for a single timepoint, colored for each subject. **(B)** Precision@150 of the cluster for each feature vector.

The distributions of the feature vectors extracted by our proposed neural network, ROI-pooling, and PCA were visualized by t-SNE, and are depicted in Figure 2A. It should be noted that t-SNE preserves local adjacence well but it does not necessarily retain the global structure.

The features extracted by the network clustered for each subject, unlike the features extracted by ROI-pooling and PCA. The clustering performance was quantitatively evaluated using precision@150 around the identity feature for each subject. The precision@150 was 81.5 and 61.4% for our proposed *classification* and *classification+* feature vectors, whereas it was 5.6% for the ROI-pooled feature and the top three and 10,000 principal components (Figure 2B). The precision@150 for each subject is shown in Supplementary Section 3.

### Experiment 3: Schizophrenia Diagnosis

**FIGURE 3:**
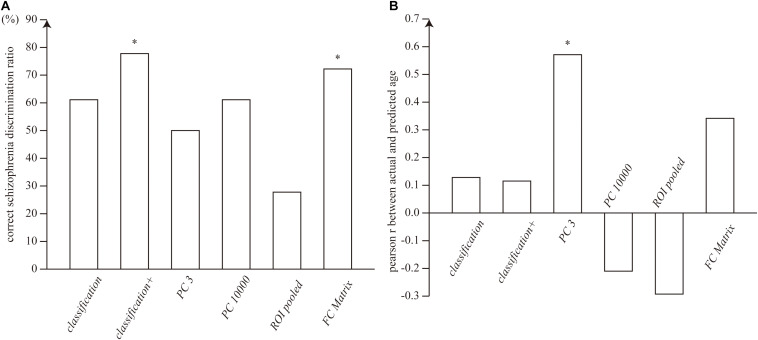
**(A)** Accuracy of the schizophrenia discrimination. **(B)** Pearson’s correlation coefficient of the age regression. The single asterisks show the statistical significance at α = 0.05.

The average of the features was computed as the identity feature for each subject, and the identity features were fed into a linear classifier for schizophrenia diagnosis with a logistic loss function. The accuracies were 61.1 and 77.8% for the identity feature of our proposed *classification* and *classification+* feature vectors, respectively. The performance of *classification+* was significantly better than the chance (*p* = 0.015). The accuracy was 72.2% for the connectivity matrix, which was marginally higher above the chance (*p* = 0.048). The identity features of the top three and 10,000 principal components and the ROI-pooled signals did not significantly discriminate between the schizophrenia and control group (acc. = 27.8, 50, and 61.1%, respectively), as shown in Figure 3A.

Similarly, subject age was regressed from the identity feature. The correlations between the predicted and actual age were not significant (*r* = 0.128 and 0.115 for *classification* and *classification+*), while the top three principal components showed significant correlation (*r* = 0.57, *p* = 0.013). The other conditions (i.e., the top 10,000 principal components, ROI-pooled signals, and functional connectivity matrix) did not show significant correlation (*r* = −0.21, −0.29, and 0.34, respectively), as shown in Figure 3B.

## Discussion

We have shown that: (i) the self-supervised learning scheme led our neural network to acquire the projection from the high (∼10^6^) dimensional signal space to the lower dimensional (∼10^2^) feature space in which each dimension represented subject identity in the training dataset, (ii) the capability of the subject identification was generalized to the unknown subjects in the test dataset, and (iii) the temporal average of the extracted feature vector reflected the psychiatric status of the subjects. Surprisingly, our proposed method performed comparable to or even better than the functional connectivity matrix for schizophrenia diagnosis, which has been regarded as a promising biomarker of cognitive functions (Liang et al., 2006; Kim et al., 2016) and reported to reflect the cognitive trait in subjects (Finn et al., 2015).

The transferred capability from the subject identification to schizophrenia diagnosis can be regarded as a kind of “deep feature extraction.” In the natural scene image processing, the intermediate output in a neural network pre-trained with a large-dataset classification often works well in another task, known as a “deep feature extraction” (Oquab et al., 2014). The underlying mechanism of the transferability is still under debate; however, one of the dominant hypotheses is that the stacked two-dimensional convolution itself works as the statistical prior of the natural scene images, regardless of the training task (Ulyanov et al., 2018). Our results showed that the transference also occurred with the combination of the human-brain T2^∗^ images and the stacked three-dimensional convolutions.

Our feature did not correlate with the subject’s age, unlike the psychiatric status. This result suggests that subjects with similar psychiatric status are adjacent on the feature space, whereas similar age subjects are not. Given this discussion, the linear-decomposition-based features (i.e., the principal/independent components) and the functional connectivity matrix might have potentially ignored the discontinuity on the signal-space, yielding the results in the subject’s age regression different from our identity feature.

Our identity feature and the functional connectivity exhibited a significant performance on schizophrenia diagnosis. The functional connectivity has been reported to be a good subject identifier (Finn et al., 2015), and thus, the features that classified patients from controls were those which behaved as the identifier of the subjects. The linkage between subject identification and the subject’s mental condition should be investigated in future works. Although the difference in diagnosis accuracy was not statistically tested due to the shortage of samples, the diagnosis accuracy of our identity feature was slightly greater than that of the functional connectivity. A potential reason behind this superiority is the local interactions of the signal. In the functional connectivity analysis, the signals are averaged for each ROI, discarding the local signal interactions. In contrast, previous studies have reported that both global and local activities in the brain lead to our cognitive functions (see Panzeri et al., 2015 for review). Both of the local and global interactions are modeled in the neural network, and it might have led to a positive effect in schizophrenia discrimination.

We introduced two versions of identity feature in this study, namely “classification” and “classification+.” Both the “classification” and “classification+” feature vectors are the intermediate output of our neural network but the characteristics of these feature vectors were slightly different: “classification” feature vectors clustered more cohesive around the subject’s identity feature than “classification+” feature vectors, while “classification+” identity feature performed better for schizophrenia diagnosis. The better performance of “classification+” in the schizophrenia diagnosis might be attributed to the small training dataset. The “classification+” feature can be regarded as the projected space from the “classification” feature space to a hyperplane tangential to **1** (vector of all ones), which reduces the degree of freedom and potentially regularizes the feature space. The regularization of the feature space might have positively affected the training with small samples for the schizophrenia diagnosis. The relation between these two types of feature vectors should be investigated in future work with a larger dataset.

In this study, we introduced a novel self-supervised learning scheme and highlighted some of the characteristics of the extracted feature, especially in terms of the relation to schizophrenia. A few parameters, such as the optimal number of subjects in the training, the optimal neural network architecture, more detailed relations between the feature and the subject’s attributes, and the mathematical analyses about the feature space will be addressed in the future work. Furthermore, for the clinical application, it is essential to evaluate the diagnosis accuracy and robustness more precisely with larger dataset as well as to explore better regressors rather than a simple linear regressor. We hope these will be uncovered in future works along the further accumulation of available datasets and with the advancement in the field of machine learning.

## Data Availability Statement

Publicly available datasets were analyzed in this study. This data can be found here: http://fcon_1000.projects.nitrc.org/indi/retro/cobre.html, the Center for Biomedical Research Excellence.

## Ethics Statement

The studies involving human participants were reviewed and approved by the Committee on Medical Ethics of the National Center of Neurology and Psychiatry. Written informed consent for participation was not required for this study in accordance with the national legislation and the institutional requirements.

## Author Contributions

YH and YY conceived and designed the research and drafted the manuscript. YH conducted the experiments and analyzed the data. YO supported preprocessing of MRI data. YO and MH provided critical revisions. All authors contributed to and have approved the final manuscript.

## Conflict of Interest

The authors declare that the research was conducted in the absence of any commercial or financial relationships that could be construed as a potential conflict of interest.

## Publisher’s Note

All claims expressed in this article are solely those of the authors and do not necessarily represent those of their affiliated organizations, or those of the publisher, the editors and the reviewers. Any product that may be evaluated in this article, or claim that may be made by its manufacturer, is not guaranteed or endorsed by the publisher.
