# Supplementary material for: Deep Feature Extraction for Resting-State Functional MRI by Self-Supervised Learning and Application to Schizophrenia Diagnosis
Source: Front Neurosci. 2021 Aug 27;15:696853. doi: 10.3389/fnins.2021.696853 (PMC8429808; doi:10.3389/fnins.2021.696853)
Supplement: Supplementary file 1 [file Table_1.DOCX]

Supplementary Material

# Preprocessing of functional MRI

Each scan was spatially realigned, timing-corrected, segmented, normalized to the Montreal Neurological Institute (MNI) coordinates, and smoothened by the default preprocessing pipeline of the CONN toolbox (Whitfield-Gabrieli and Nieto-Castanon, 2012). The resulting spatial resolution was 91 × 109 × 91 voxels. Subsequently, its default denoising pipeline was applied. We masked out the voxels of white matter and cerebrospinal tissue and acquired a gray matter mask of 150,571 voxels. We then normalized the signal intensity within the gray matter mask for each scan to prevent the network from learning the bias and variance as a clue for subject classification; the signals out of the gray-matter mask were replaced by zero.

# Formula for precision@K

$$precision@K := \frac{\sum_{s=1}^{N_{sbj}} prec_{s}}{N_{sbj}}$$

$${prec}_{s}:=\frac{\sum_{k=1}^{K} \boldsymbol{1}_{A_{s}}\left( x_{sk} \right)}{K}$$

$$x_{sk}:=(the k\text{-}th nearest x to the {centroid}_{s})$$

$${centroid}_{s}:= \frac{\sum_{x\in A_{s}} x}{N_{frame}}$$

$$\boldsymbol{1}_{A}\left( x \right):=\left\{ \begin{aligned} 1 if x\in A \\ 0 if x\notin A \end{aligned} \right.$$

$$A_{s}:=(x_{i} tied to subject s)$$

# Precision@150 for each subject ID

| Subject ID | Classification | Classification+ |
| --- | --- | --- |
| Subject #1 | 1.00 | 0.83 |
| Subject #2 | 1.00 | 0.67 |
| Subject #3 | 0.72 | 0.67 |
| Subject #4 | 0.83 | 0.83 |
| Subject #5 | 0.67 | 0.44 |
| Subject #6 | 0.83 | 0.61 |
| Subject #7 | 0.61 | 0.67 |
| Subject #8 | 1.00 | 0.89 |
| Subject #9 | 0.83 | 0.67 |
| Subject #10 | 1.00 | 0.67 |
| Subject #11 | 0.72 | 0.17 |
| Subject #12 | 0.72 | 0.28 |
| Subject #13 | 0.94 | 0.56 |
| Subject #14 | 0.67 | 0.56 |
| Subject #15 | 1.00 | 1.00 |
| Subject #16 | 0.83 | 0.61 |
| Subject #17 | 0.61 | 0.50 |
| Subject #18 | 0.67 | 0.44 |

# References

Whitfield-Gabrieli, S., and Nieto-Castanon, A., (2012). Conn: A Functional Connectivity Toolbox for Correlated and Anticorrelated Brain Networks. Brain Connect. 2:3, 125–141.
